# Prevalence of Endodontic-Periodontal Lesions and Associated Risk Factors Among Patients Undergoing Orthodontic Treatment: A Retrospective Observational Study

**DOI:** 10.7759/cureus.115639

**Published:** 2026-09-02

**Authors:** Aishwarya P Dahikar, Anshita Shrivastava, Sunitha Kaggal, Chandana LR, Jay Gohil, Grishmi Niswade

**Affiliations:** 1 Department of Orthodontics and Dentofacial Orthopedics, Ranjeet Deshmukh Dental College and Research Centre, Nagpur, IND; 2 Department of Periodontics, Modern Dental College and Research Centre, Indore, IND; 3 Department of Orthodontics and Dentofacial Orthopedics, Sri Hasanamba Dental College and Hospital, Hassan, IND; 4 Department of Orthodontics and Dentofacial Orthopedics, Educare Institute of Dental Sciences, Malappuram, IND; 5 Department of Prosthodontics, K. M. Shah Dental College and Hospital, Sumandeep Vidyapeeth (Deemed to Be University), Vadodara, IND; 6 Department of Periodontics, Swargiya Dadasaheb Kalmegh Smruti Dental College and Hospital, Nagpur, IND

**Keywords:** endodontic-periodontal lesions, orthodontic treatment, periodontal disease, prevalence, pulp vitality, retrospective study, risk factors

## Abstract

Introduction: Endodontic-periodontal (endo-perio) lesions are complex pathological conditions involving simultaneous pulpal and periodontal tissue destruction, posing considerable diagnostic and therapeutic challenges. Although orthodontic treatment may influence periodontal health, limited evidence exists regarding the prevalence of endo-perio lesions and their associated risk factors among orthodontic patients. This study aimed to determine the prevalence of endo-perio lesions among patients undergoing orthodontic treatment and to identify demographic, clinical, and radiographic factors associated with their occurrence.

Materials and methods: This retrospective observational study analyzed archived records of orthodontic patients. During the six-month record-review period, 1,250 orthodontic patient records were screened. Diagnosis was established using documented clinical findings, pulp vitality testing, and radiographic evaluation. Data were then analyzed, and continuous variables were compared using the independent-samples t test or Mann-Whitney U test, whereas categorical variables were compared using the chi-square test or Fisher's exact test. Independent predictors were identified using multivariable logistic regression. Statistical significance was established at p < 0.05.

Results: The overall prevalence of endo-perio lesions was 5.76% (72/1,250). Patients with lesions were significantly older than those without lesions (33.7 ± 9.8 vs. 28.1 ± 9.0 years; p < 0.001) and had a longer median orthodontic treatment duration (27.0 vs. 24.0 months; p = 0.009). Multivariable logistic regression identified that increasing age (adjusted OR = 1.04; 95% CI: 1.01-1.08; p = 0.012), molar teeth (adjusted OR = 2.87; 95% CI: 1.20-6.84; p = 0.017), non-vital pulp (adjusted OR = 52.90; 95% CI: 20.72-135.10; p < 0.001), deep periodontal pockets ≥5 mm (adjusted OR = 92.14; 95% CI: 21.80-389.50; p < 0.001), and radiographic bone loss (adjusted OR = 18.47; 95% CI: 5.46-62.52; p < 0.001) were independent predictors of endo-perio lesions.

Conclusion: Older age, molar teeth, non-vital pulp, deep periodontal pockets, and radiographic bone loss were significant independent predictors of lesion occurrence. Comprehensive periodontal examination, pulp vitality testing, and radiographic assessment should be routinely integrated into orthodontic care to facilitate early diagnosis and interdisciplinary management.

## Introduction

Endodontic-periodontal (endo-perio) lesions represent a challenging clinical condition characterized by the simultaneous involvement of the dental pulp and the supporting periodontal tissues. Because the pulp and periodontium are interconnected through anatomical pathways such as the apical foramen, lateral and accessory canals, and dentinal tubules, disease processes originating in one tissue can extend to the other, resulting in complex lesions that often complicate diagnosis and treatment [1,2]. Accurate differentiation between primary endodontic, primary periodontal, and true combined lesions is essential, as management strategies and treatment outcomes differ considerably depending on the origin and extent of the disease [3]. Despite advances in diagnostic techniques, including pulp vitality testing and three-dimensional imaging, distinguishing these lesions remains a significant clinical challenge [1,4].

Orthodontic treatment introduces biological changes within the periodontal ligament and surrounding alveolar bone through the application of controlled mechanical forces [5]. While these changes are generally physiological, prolonged orthodontic therapy, inadequate oral hygiene, plaque accumulation, excessive orthodontic forces, or pre-existing pulpal and periodontal pathology may increase the susceptibility to endo-perio complications [6]. Early recognition of such lesions during orthodontic treatment is crucial because delayed diagnosis may compromise tooth prognosis, prolong orthodontic treatment, and adversely affect overall clinical outcomes. However, there is limited epidemiological evidence regarding the prevalence of endo-perio lesions among orthodontic patients, particularly in retrospective institutional cohorts.

The aim of this study was to determine the prevalence of endo-perio lesions among orthodontic patients referred for periodontal evaluation and to evaluate the demographic, clinical, radiographic, and treatment-related factors associated with their occurrence using retrospective institutional records. Secondary objectives were to describe the distribution of endo-perio lesion types and their clinical and radiographic characteristics and to determine the independent predictors of endo-perio lesions using multivariable logistic regression analysis.

## Materials and methods

This retrospective observational study was conducted in the Department of Periodontics, Swargiya Dadasaheb Kalmegh Smruti Dental College and Hospital, Nagpur, India. During the six-month record-review period from April to September 2024, 1,250 orthodontic patient records from January 2012 to February 2024 were screened. Since the investigation involved analysis of previously documented clinical records, no intervention or modification of patient treatment was performed. The study involved retrospective analysis of anonymized patient records without direct patient interaction, and ethical approval was obtained from the Institutional Ethics Committee (SDKSDCH/IEC/2024/112 dated March 20, 2024). All patient information was de-identified before data extraction, and confidentiality was maintained throughout the study in accordance with the Declaration of Helsinki and institutional policies regarding data protection.

The institutional database containing records of patients who had undergone fixed orthodontic treatment between January 2012 and February 2024 served as the sampling frame. During this period, all patients referred from the Department of Orthodontics to the Department of Periodontology for periodontal evaluation and management were identified through the institutional referral registry. A total of 1,250 orthodontic patient records were screened for eligibility. Archived clinical records, periodontal examination charts, endodontic records, digital intraoral periapical radiographs, and orthopantomograms (OPGs) were comprehensively reviewed to identify endo-perio lesions. Following application of the predefined eligibility and diagnostic criteria, 72 patients fulfilled the inclusion criteria and were included in the final analysis.

Patients were included if they had received fixed orthodontic treatment, were referred for periodontal evaluation, possessed complete clinical records, including periodontal examination findings, endodontic assessment, and radiographic investigations, and had a definitive diagnosis of an endo-perio lesion documented by qualified specialists. Patients with incomplete records, teeth exhibiting traumatic injuries, vertical root fractures, root perforations, external cervical resorption, systemic conditions known to significantly influence periodontal healing, duplicate records, or teeth extracted before complete diagnostic evaluation were excluded from the study.

Diagnosis and classification of endo-perio lesions were established through a comprehensive evaluation of archived clinical records from the departments of endodontics and periodontology. Eligible cases were retrospectively classified according to the diagnostic framework of the 2017 World Workshop Classification of Periodontal and Peri-implant Diseases and Conditions [7]. The assessment incorporated documented periodontal findings, including periodontal probing findings, clinical attachment loss, tooth mobility, gingival swelling or sinus tract, and furcation involvement; endodontic findings, including pulp vitality test results and previous endodontic treatment; and radiographic findings, including periapical or lateral radiolucency and periodontal, periapical, or combined bone loss. Cases were classified as primary endodontic, primary periodontal, or true combined endo-perio lesions according to the predominant clinical, endodontic, and radiographic findings documented in the records. Primary endodontic lesions were characterized by findings indicating a predominant endodontic origin, whereas primary periodontal lesions were characterized by findings indicating a predominant periodontal origin. True combined endo-periodontal lesions were identified when clinical, endodontic, and radiographic findings demonstrated concurrent endodontic and periodontal involvement. Cases were included only when sufficient clinical, endodontic, and radiographic information was available to support classification; records with insufficient diagnostic information were excluded.

Radiographic assessment was performed using the available digital intraoral periapical radiographs and OPGs. Radiographs were reviewed for periapical or lateral radiolucency, periodontal bone loss, vertical bone loss, furcation involvement, and combined periodontal and periapical bone loss, with assessment of the extent and characteristics of osseous destruction and evidence of pulpal-periodontal communication. Cone-beam CT (CBCT) findings, when available, were considered as supplementary diagnostic information but were not required for inclusion.

Data extraction was performed independently by two calibrated investigators (AS, CLR). Variables recorded included age, sex, type and duration of orthodontic treatment, tooth involved, arch involved, tooth type, periodontal findings, endodontic diagnosis, radiographic characteristics, and presence or absence of endo-perio lesions. Following independent data collection, all extracted data were verified by a third investigator (GN) to minimize transcription errors, and any discrepancies were resolved by consensus after re-examination of the original patient records. Prior to data extraction, two investigators independently evaluated a randomly selected set of patient records that were not included in the final study sample to assess examiner reliability. Interexaminer agreement for the diagnosis of endo-perio lesions was assessed using Cohen's kappa (κ) coefficient, which demonstrated excellent agreement (κ = 0.89; 95% CI: 0.83-0.95). The high κ value indicated excellent reproducibility between the examiners, and therefore no further calibration was required before commencement of full-scale data extraction.

The primary outcome measure was the prevalence of endo-perio lesions among orthodontic patients referred for periodontal evaluation. Secondary outcome measures included the distribution of lesion types, clinical and radiographic characteristics of the lesions, and demographic and treatment-related factors associated with their occurrence. Prevalence was calculated using the following formula:

\[ \text{Prevalence (%)} = \left( \frac{\text{Number of patients diagnosed with endo-perio lesions}} {\text{Total number of orthodontic patients screened}} \right) \times 100 \]

Statistical analysis was performed using IBM SPSS Statistics for Windows, Version 26.0 (IBM Corporation, Armonk, NY, USA). The normality of continuous variables was assessed using the Shapiro-Wilk test. Continuous variables with normal distribution were expressed as mean ± standard deviation (SD), whereas non-normally distributed variables were presented as median and interquartile range (IQR). Categorical variables were summarized as frequencies and percentages. The prevalence of endo-perio lesions was calculated with corresponding 95% CI. Associations between categorical variables were evaluated using the chi-square test or Fisher's exact test, as appropriate, while continuous variables were compared using the independent samples t-test for normally distributed data or the Mann-Whitney U test for non-normally distributed data. Variables demonstrating statistical significance in the univariate analysis were subsequently entered into a multivariable binary logistic regression model to identify independent predictors of endo-perio lesions after adjustment for potential confounding variables. Adjusted ORs with 95% CIs were reported. The goodness-of-fit of the regression model was evaluated using the Hosmer-Lemeshow test, Nagelkerke R², and −2 log likelihood statistics. All statistical tests were two-tailed, and a p-value < 0.05 was considered statistically significant.

## Results

A total of 1,250 orthodontic patient records were screened, and 72 (5.76%) patients were diagnosed with endo-perio lesions. Patients with endo-perio lesions had a significantly higher mean age than those without endo-perio lesions (33.7 ± 9.8 years vs. 28.1 ± 9.0 years; p < 0.001). Although female sex was significantly associated with lesion occurrence in the univariate analysis (p = 0.016), it was not an independent predictor in the multivariable model. Patients diagnosed with endo-perio lesions also demonstrated a significantly longer median duration of orthodontic treatment than patients without lesions (27.0 vs. 24.0 months; p = 0.009). Molar teeth were affected more frequently than other tooth types (p = 0.020), while mandibular involvement was relatively more common among patients with lesions. Clinical findings including non-vital pulp, periodontal pockets ≥5 mm, radiographic bone loss, and furcation involvement were significantly more prevalent among patients with endo-perio lesions compared with those without lesions (all p < 0.001) (Table 1).

**Table 1:** Demographic and clinical characteristics of the study population (N = 1,250). Data are presented as mean ± standard deviation (SD), median (interquartile range (IQR)), or frequency (percentage). Continuous variables were compared using the independent samples t-test for normally distributed variables or the Mann–Whitney U test for non-normally distributed variables. Categorical variables were compared using the chi-square (χ²) test. Effect sizes are reported as Cohen's d, Phi coefficient (φ), Cramer's V, or r, A two-tailed *p < 0.05 was considered statistically significant. t: independent samples t-test; U: Mann–Whitney U test; df: degrees of freedom.

Characteristic		Total (N = 1,250)	Patients without endo-perio lesion (n = 1,178)	Patients with endo-perio lesion (n = 72)	Test statistic (df)	p-value	Effect size
Age, Mean ± SD	Years	28.4 ± 9.1	28.1 ± 9.0	33.7 ± 9.8	t(1248) = -5.12	< 0.001*	Cohen's d = 0.59
Sex, n (%)	Male	578 (46.2)	548 (46.5)	30 (41.7)	χ²(1) = 5.82	0.016*	φ = 0.068
	Female	672 (53.8)	630 (53.5)	42 (58.3)			
Orthodontic treatment duration, Median (IQR)	Months	24.0 (18.0 - 30.0)	24.0 (18.0 - 29.0)	27.0 (21.0 - 34.5)	U = 32,451.5	0.009*	r = 0.073
Arch involved, n (%)	Maxillary	712 (57.0)	676 (57.4)	36 (50.0)	χ² (1) = 15.05	< 0.001*	φ = 0.110
	Mandibular	538 (43.0)	502 (42.6)	36 (50.0)			
Tooth type, n (%)	Incisor/canine	612 (49.0)	587 (49.8)	25 (34.7)	χ² (3) = 9.83	0.020*	Cramer's V = 0.089
	Premolar	488 (39.0)	455 (38.6)	33 (45.8)			
	Molar	150 (12.0)	136 (11.5)	14 (19.4)			
Pulp vitality, n (%)	Negative	180 (14.4)	115 (9.8)	65 (90.3)	χ² (1) = 398.4	< 0.001*	φ = 0.565
Presence of deep pockets, n (%)	≥5 mm	310 (24.8)	240 (20.4)	70 (97.2)	χ² (1) = 242.8	< 0.001*	φ = 0.441
Radiographic bone loss, n (%)	Yes	425 (34.0)	356 (30.2)	69 (95.8)	χ² (1) = 129.0	< 0.001*	φ = 0.321
Furcation involvement, n (%)	Yes	98 (7.8)	84 (7.1)	14 (19.4)	χ² (1) = 15.38	< 0.001*	φ = 0.111

Increasing age was significantly associated with the occurrence of endo-perio lesions, with each additional year increasing the odds by approximately 6% (OR = 1.06; 95% CI: 1.04-1.09; p < 0.001). Longer orthodontic treatment duration also showed a significant association (OR = 1.04; 95% CI: 1.01-1.07; p = 0.004). Compared with incisors and canines, molar teeth were associated with significantly greater odds of developing endo-perio lesions (OR = 2.41; 95% CI: 1.20-4.86; p = 0.014). The strongest associations were observed for non-vital pulp (OR = 81.09), deep periodontal pockets ≥5 mm (OR = 136.04), and radiographic bone loss (OR = 66.71), all of which were highly significant (p < 0.001). Furcation involvement was also significantly associated with lesion occurrence (OR = 3.15; p < 0.001), whereas sex and arch involvement did not demonstrate statistically significant associations (Table 2).

**Table 2:** Univariate logistic regression analysis of factors associated with endodontic-periodontal lesions. Logistic regression analysis was performed to evaluate the association between individual variables and endodontic-periodontal lesions. A two-tailed *p < 0.05 was considered statistically significant. Reference (Ref.) categories are indicated for categorical variables.

Variable	Category	Odds ratio (unadjusted)	95% confidence interval	p-value
Age	(Per 1-year increase)	1.06	1.04 - 1.09	< 0.001*
Sex	Female (Ref: Male)	1.22	0.75 - 1.98	0.416
Treatment duration	(Per 1-month increase)	1.04	1.01 - 1.07	0.004*
Arch involved	Mandibular (Ref: Maxillary)	1.34	0.84 - 2.16	0.218
Tooth type	Premolar (Ref: Incisor/Cuspid)	1.70	1.00 - 2.90	0.050
	Molar (Ref: Incisor/Cuspid)	2.41	1.20 - 4.86	0.014*
Pulp vitality	Non-vital (Ref: Vital)	81.09	35.62 - 184.60	< 0.001*
Deep pockets (≥5 mm)	Present (Ref: Absent)	136.04	33.43 - 553.50	< 0.001*
Radiographic bone loss	Present (Ref: Absent)	66.71	21.25 - 209.40	< 0.001*
Furcation involvement	Present (Ref: Absent)	3.15	1.70 - 5.85	< 0.001*

After adjustment for potential confounding variables, the multivariable logistic regression model identified several independent predictors of endo-perio lesions. Age remained a significant predictor, with each one-year increase associated with a 4% increase in the odds of lesion occurrence (adjusted OR = 1.04; 95% CI: 1.01-1.08; p = 0.012). Molar teeth continued to demonstrate significantly higher odds compared with incisors and canines (adjusted OR = 2.87; 95% CI: 1.20-6.84; p = 0.017). The strongest independent predictors were non-vital pulp (adjusted OR = 52.90), deep periodontal pockets ≥5 mm (adjusted OR = 92.14), and radiographic bone loss (adjusted OR = 18.47), all of which remained highly significant (p < 0.001). Orthodontic treatment duration and furcation involvement were no longer statistically significant after adjustment. The logistic regression model demonstrated excellent calibration (Hosmer-Lemeshow p = 0.410) and indicated substantial explanatory performance (Nagelkerke R² = 0.68) (Table 3).

**Table 3:** Multivariable logistic regression analysis identifying independent predictors of endodontic-periodontal lesions. Variables with statistical significance in the univariate analysis were entered into the multivariable logistic regression model. Model performance was assessed using the Hosmer–Lemeshow goodness-of-fit test, Nagelkerke R², and −2 log likelihood. A two-tailed *p < 0.05 was considered statistically significant Model fit: The multivariable logistic regression model demonstrated good overall fit (−2 log likelihood = 345.82; Hosmer–Lemeshow χ² = 8.24, p = 0.410), explained 68% of the variance in endodontic-periodontal lesion occurrence (Nagelkerke R² = 0.68), and was statistically significant overall (p < 0.001). R²: coefficient of determination

Variable	Category	Adjusted odds ratio	95% confidence interval	p-value	Effect size
Age	(Per 1-year increase)	1.04	1.01 - 1.08	0.012*	0.04
Treatment duration	(Per 1-month increase)	1.03	1.00 - 1.06	0.089	0.02
Tooth type	Molar (Ref: Incisor/Cuspid)	2.87	1.20 - 6.84	0.017*	0.06
Pulp vitality	Non-vital (Ref: Vital)	52.90	20.72 - 135.1	< 0.001*	0.35
Deep pockets (≥5 mm)	Present (Ref: Absent)	92.14	21.80 - 389.5	< 0.001*	0.22
Radiographic bone loss	Present (Ref: Absent)	18.47	5.46 - 62.52	< 0.001*	0.11
Furcation involvement	Present (Ref: Absent)	1.98	0.83 - 4.72	0.124	0.01

Primary periodontal lesions represented the most common diagnostic category, accounting for 32 (44.4%) of cases, followed by primary endodontic lesions and true combined lesions, each comprising 20 (27.8%) of the study sample. Radiographically, combined periapical and vertical bone loss was the most frequent presentation, followed by isolated vertical bone loss and isolated periapical radiolucency. Regarding tooth mobility, Grade II mobility was the most common finding, followed by Grade I and Grade III mobility. A sinus tract or gingival swelling was present in 25 (34.7%) of patients. Pulp vitality testing demonstrated that 65 (90.3%) of affected teeth were non-vital, whereas only seven (9.7%) retained pulpal vitality, emphasizing the close relationship between pulpal necrosis and endo-perio lesions in orthodontic patients (Table 4).

**Table 4:** Clinical and radiographic characteristics of patients with confirmed endodontic-periodontal lesions (n = 72). Data are presented as frequency (percentage). Percentages were calculated using the total number of patients with confirmed endodontic-periodontal lesions (n = 72).

Diagnostic parameter	Category	Frequency, n (%)
Primary diagnosis classification	Primary endodontic	20 (27.8)
	Primary periodontal	32 (44.4)
	Combined true lesion	20 (27.8)
Type of bone loss (radiographic)	No bone loss	3 (4.1)
	Periapical radiolucency only	12 (16.7)
	Isolated vertical bone loss	28 (38.9)
	Combined periapical and vertical bone loss	29 (40.3)
Tooth mobility (Miller's Grade)	Grade I	28 (38.9)
	Grade II	30 (41.7)
	Grade III	14 (19.4)
Sinus tract/swelling	Present	25 (34.7)
	Absent	47 (65.3)
Pulp vitality test result	Vital	7 (9.7)
	Non-vital	65 (90.3)

## Discussion

The present retrospective observational study evaluated the prevalence of endo-perio lesions among patients undergoing orthodontic treatment and identified demographic, clinical, and radiographic factors associated with their occurrence. Among 1,250 orthodontic patients screened, 72 patients were diagnosed with endo-perio lesions, resulting in an overall prevalence of 5.76%. This finding indicates that although endo-perio lesions are relatively uncommon in orthodontic practice, they represent an important clinical entity because of their complex diagnosis and the need for coordinated management between orthodontists, endodontists, and periodontists. A comparably low prevalence of endo-perio lesions has been reported by Sarı and Altındal [8], who observed that true endo-perio lesions constituted only a small proportion of patients presenting with periodontal disease. Their findings support the concept that these lesions are relatively infrequent but often associated with extensive tissue destruction and poorer prognosis when diagnosis is delayed.

Similarly, Rotstein and Simon [9] emphasized that true combined lesions are uncommon compared with isolated endo-perio lesions but require meticulous differential diagnosis because treatment depends on identifying the primary source of infection. In contrast, Shiggaon et al. [10] reported a substantially higher prevalence of 29.49% in an Indian cohort. The difference is expected because the present investigation was restricted to patients undergoing orthodontic treatment who were referred for periodontal evaluation, whereas the authors evaluated a broader adult dental population with established endo-perio lesions. Differences in study population, diagnostic criteria, and sampling methodology may also have contributed to the variation in prevalence.

A significant finding of the present study was that patients with endo-perio lesions were significantly older than unaffected individuals, and increasing age remained an independent predictor after multivariable analysis. This observation is consistent with previous reports showing that periodontal destruction and pulpal degeneration increase with age. Sarı and Altındal [8] similarly demonstrated a higher prevalence of endo-perio lesions among older adults, suggesting that cumulative periodontal attachment loss, restorative procedures, calcification of root canals, and reduced pulpal vascularity contribute to greater susceptibility. This finding agrees with Shiggaon et al. [10], who also reported a significant association between age and endo-perio lesions. Age-related reductions in immune response and prolonged exposure to microbial biofilms may further explain the increased risk observed in older orthodontic patients [11].

Orthodontic treatment duration was significantly longer among patients with endo-perio lesions; however, it did not remain an independent predictor after adjustment for confounding factors. This finding suggests that prolonged orthodontic treatment itself may not directly induce endo-perio lesions but rather reflects prolonged plaque accumulation, oral hygiene challenges, and persistence of pre-existing periodontal disease [12]. A previous systematic review evaluating periodontal health during orthodontic treatment has similarly reported that fixed appliances increase plaque retention and gingival inflammation when oral hygiene is inadequate [13]. Nevertheless, most investigators have concluded that orthodontic treatment alone does not permanently damage the periodontium in patients receiving appropriate periodontal maintenance, which is in agreement with the present findings [14].

The present study also demonstrated that molar teeth were associated with significantly greater odds of endo-perio lesions than incisors or canines. Similar observations have been reported in epidemiological studies evaluating endo-perio lesions, where molars consistently represented the most frequently affected teeth because of their complex root canal anatomy, multiple accessory canals, furcation regions, and greater susceptibility to both caries and periodontal destruction [15,16]. The presence of accessory canals within furcation areas provides direct communication between pulpal and periodontal tissues, facilitating microbial spread in both directions [16]. Furthermore, molars are generally more difficult for orthodontic patients to clean effectively because of orthodontic bands and their posterior location, thereby increasing biofilm accumulation and periodontal inflammation [17].

Among all evaluated variables, non-vital pulp demonstrated one of the strongest associations with endo-perio lesions. More than 90% of affected teeth were non-vital, and this variable remained an independent predictor in the logistic regression model. These findings are consistent with the classic biological concepts proposed by Simon et al. [3], who described pulpal necrosis as one of the principal initiating factors for endo-perio lesions. Similarly, Rotstein and Simon [9] reported that microorganisms originating from infected root canal systems migrate through the apical foramen, lateral canals, and dentinal tubules into surrounding periodontal tissues, producing inflammatory destruction. Conversely, advanced periodontal destruction may also extend toward the pulp through accessory canals, explaining the bidirectional relationship observed in advanced lesions [18]. The high frequency of non-vital teeth observed in the present study, therefore, strongly supports the established pathophysiology of endo-perio lesions.

Deep periodontal pockets and radiographic bone loss were the strongest independent clinical predictors identified in this investigation. These findings closely resemble those reported by Sarı and Altındal [8], who identified periodontal pocket depth and radiographic bone destruction as the most consistent clinical indicators associated with endo-perio lesions. Deep periodontal pockets facilitate microbial migration toward accessory canals, while extensive vertical bone loss compromises periodontal attachment and increases the possibility of pulpal involvement [18]. Radiographically, combined periapical and vertical bone defects were the most frequent presentation in the present study, further supporting previous reports that simultaneous pulpal and periodontal involvement often produces characteristic combined osseous defects visible on radiographic examination [1,3].

Although the present study demonstrated a high frequency of non-vital pulp among teeth with endo-perio lesions, Sabeti et al. [19] reported that severe periodontitis alone did not significantly compromise pulp vitality, as all examined teeth remained vital despite extensive periodontal destruction. However, they observed significantly greater apical pulpal inflammation, pulp fibrosis, and internal and external root resorption in periodontally compromised teeth. These findings suggest that periodontal disease may be associated initially with inflammatory and degenerative pulpal changes, whereas complete pulpal necrosis may occur only after bacterial invasion through accessory pathways or concomitant endodontic infection.

Although furcation involvement demonstrated a significant association in the univariate analysis, it was no longer statistically significant after adjustment in the multivariable model. Similar findings have been reported in previous observational studies, suggesting that furcation involvement frequently reflects advanced periodontal destruction rather than serving as an independent etiological factor [8,20]. Likewise, sex was not associated with lesion occurrence, which agrees with most epidemiological studies indicating that biological factors, oral hygiene status, and periodontal condition are stronger determinants than patient sex [8,10].

The distribution of lesion types in the present study demonstrated that primary periodontal lesions were the most common, followed by primary endodontic lesions and true combined lesions. Comparable distributions have been reported by Sarı and Altındal [8] and by previous classification studies based on the 2017 World Workshop, which observed that true combined lesions occur less frequently than isolated periodontal lesions [7,20]. The predominance of primary periodontal lesions may be explained by the high prevalence of chronic periodontitis in adults and the progressive extension of periodontal destruction toward the apical region. Nevertheless, the high proportion of non-vital teeth observed in the present investigation highlights the necessity of pulp vitality testing before periodontal therapy to avoid inappropriate treatment planning [21].

Clinical implications

The present findings emphasize that comprehensive clinical examination, pulp vitality testing, periodontal probing, and radiographic assessment should be routinely performed before initiating orthodontic treatment, particularly in older patients and molar teeth with previous restorations or periodontal destruction. Early interdisciplinary collaboration among orthodontists, periodontists, and endodontists is essential for accurate diagnosis and timely management of suspected endo-perio lesions. Regular periodontal maintenance, meticulous plaque control, and periodic reassessment during orthodontic treatment may substantially reduce disease progression and improve long-term treatment outcomes.

Limitations

This study was retrospective and dependent on the completeness and accuracy of archived records. The referral-based sampling may have introduced selection and referral bias and limits the generalizability of the findings. The retrospective design also limits assessment of temporality and does not allow causal relationships to be established. Diagnostic circularity and residual confounding cannot be completely excluded because some clinical and radiographic variables used in lesion classification were also evaluated for their association with lesion occurrence. CBCT was not available for all patients, which may have limited characterization of lesion extent. Furthermore, the relatively small number of lesion cases may have resulted in model overfitting and unstable regression estimates, particularly given the large odds ratios and wide confidence intervals. Therefore, the identified factors should be interpreted as associations rather than definitive causal risk factors or validated predictors. Larger multicenter prospective studies are needed to confirm these findings.

## Conclusions

Endo-perio lesions represent clinically important conditions among orthodontic patients referred for periodontal evaluation. Increasing age, molar teeth, non-vital pulp, deep periodontal pockets, and radiographic bone loss were independently associated with the occurrence of endo-perio lesions. These findings highlight the importance of comprehensive periodontal and endodontic evaluation in orthodontic patients, with radiographic assessment performed when clinically indicated. Given the retrospective observational design, these associations should not be interpreted as causal relationships. Further prospective multicenter studies are needed to confirm these findings and clarify the temporal relationship between orthodontic treatment and endo-perio lesions.
